# Sustained postconfluent culture of human mammary epithelial cells enriches for luminal and c-Kit+ subtypes

**DOI:** 10.1186/s13058-022-01595-z

**Published:** 2023-01-18

**Authors:** Michael E. Todhunter, Masaru Miyano, Eric G. Carlson, Stefan Hinz, Mark A. LaBarge

**Affiliations:** 1 Department of Population Sciences, Beckman Research Institute at City of Hope, 1500 E. Duarte Rd, Duarte, CA 91010 USA; 2 Irell and Manella Graduate School of Biological Sciences, City of Hope, 1500 E. Duarte Rd, Duarte, CA 91010 USA

**Keywords:** Human mammary epithelial culture, Luminal, Myoepithelial, c-Kit, Postconfluent, Multilayered

## Abstract

**Background:**

A challenge in human mammary epithelial cell (HMEC) culture is sustaining the representation of competing luminal, myoepithelial, and progenitor lineages over time. As cells replicate in culture, myoepithelial cells come to dominate the composition of the culture with serial passaging. This drift in composition presents a challenge for studying luminal and progenitor cells, which are prospective cells of origin for most breast cancer subtypes.

**Methods:**

We demonstrate the use of postconfluent culture on HMECs. Postconfluent culture entails culturing HMECs for 2–5 weeks without passaging but maintaining frequent feedings in low-stress M87A culture medium. In contrast, standard HMEC culture entails enzymatic subculturing every 3–5 days to maintain subconfluent density.

**Results:**

When compared to standard HMEC culture, postconfluent culture yields increased proportions of luminal cells and c-Kit+ progenitor cells. Postconfluent cultures develop a distinct multilayered morphology with individual cells showing decreased physical deformability as compared to cells in standard culture. Gene expression analysis of postconfluent cells shows increased expression of lineage-specific markers and extracellular matrix components.

**Conclusions:**

Postconfluent culture is a novel, useful strategy for altering the lineage composition of HMECs, by increasing the proportional representation of luminal and progenitor cells. We speculate that postconfluent culture creates a microenvironment with cellular composition closer to the physiological state and eases the isolation of scarce cell subtypes. As such, postconfluent culture is a valuable tool for researchers using HMECs for breast cancer research.

**Supplementary Information:**

The online version contains supplementary material available at 10.1186/s13058-022-01595-z.

## Background

Human mammary epithelial cell (HMEC) culture is widely used to expand normal, pre-stasis, untransformed cells from human breast tissue explants. HMEC culture is possible due to specialized methods for harvesting cells from primary explants and expanding these cells in cell culture [1], allowing untransformed, normal human cells to be grown in vitro. These cultures are heterogeneous, containing a mixture of luminal epithelial cells (LEps), myoepithelial cells (MEps), and progenitor cells [2, 3]. Although HMECs can proliferate for up to 60 population doublings in appropriate culture media [4], the composition of the cells drifts over passage, with MEps eventually dominating the cultures [5], probably due to a combination of better myoepithelial adaptation to culture and lineage conversion [6]. Media choice in standard subconfluent culture is a determinant of lineage diversity, with more stressful media, defined as media that prematurely triggers the expression of p16 and limits cell replication, driving primary HMECs to monoculture within two to three passages [4]. This distorts the proportions of the cell types in the culture and diminishes the feasibility of isolating LEps and progenitor cells, which are important for experiments involving e.g. cell–cell interactions, the stem cell hierarchy, or cancer initiation. Although the lineage composition of HMEC cultures can be changed by reducing the stiffness of their culture substrate [7], this tends to reduce or abolish proliferation of the cells, as seen in 3D cultures of normal HMECs [8].

Postconfluent cell culture is a technique wherein cells are maintained at a density high enough to form a contiguous sheet. Postconfluent cell culture entails cells having prolonged contact with their neighbors, which generally leads to contact inhibition of proliferation [9]. Many factors, such as substrate stiffness [10], the concentration of mitogens [11], and adhesivity proteins such as mucins [12] and cadherins [13, 14], affect contact inhibition of epithelial cells. Under certain conditions, epithelial cells can proliferate in postconfluent conditions to form multilayered cultures [15]; among mammary epithelial cultures, the MCF-7 human mammary carcinoma cell line can grow in this manner [16, 17]. We are unaware of any reports of normal (non-cancerous) cells growing in a multilayered postconfluent manner, despite the presence of bilayered (e.g., the mammary and prostate glands) and many-layered epithelial tissues (e.g., the sebaceous and meibomian glands) in human physiology.

Here, we demonstrate postconfluent multilayered culture of normal primary HMECs. Cells are held at postconfluent density for 1–5 weeks. During this period, the cells slowly proliferate into a distinctive multilayered culture. Concomitantly, the proportion of LEps and c-Kit+ cells, which are putative progenitor cells, in the culture increases at the expense of myoepithelial cells. The abundance of LEps and luminal progenitor cells in postconfluent culture far exceeds that of our standard subconfluent cultures. Mechanical characterization shows reduced physical deformability of postconfluent cultures as compared to standard cultures, whereas gene expression profiling by RNA-seq shows characteristics intermediate between standard cultures and uncultured epithelial organoid specimens.

## Methods

### General materials and reagents

Reagents were used as received without further purification or modification.

### Cell culture

Finite-lifespan HMECs were provided by the Human Mammary Epithelial Cell (HMEC) Bank [1]. For standard 2D culture, primary human mammary epithelial cells at the 4th passage were established and maintained in M87A medium as previously described [18, 19] with the media recipe delineated in Table 1. Mycoplasma testing was performed prior to all experiments in this study. Collagen-coated polyacrylamide culture dishes were purchased from Cell Guidance Systems (Petrisoft). Specimens were used in experiments as summarized in Table 2.

**Table 1:** M87A media recipe. Combine equal volumes of MEBM and DMEM/F12 basal media, then add the supplements as listed in this table. Finally, filter-sterilize with 0.2-μm polyethersulfone membrane

Factor	Amount
Glutamine	2.0 mM
Fetal bovine serum	0.25%
Bovine pituitary extract	35 μg/mL
Insulin	3.0 mg/mL
Isoproterenol	5 μM
Hydrocortisone	0.3 μg/mL
Apo-transferrin	2.5 μg/mL
Oxytocin	0.1 nM
Cholera toxin	0.5 ng/mL
Epidermal growth factor	5.0 ng/mL
β-estradiol	500 pM
Tri-iodo-thyronine	5 nM
Alubmax I	0.1%

**Table 2:** Human mammary epithelial cell specimens used

Specimen	Age (years)	Source	Flow?	Immunofluorescence?	mNPS?	RNA-seq?
240L	19	Reduction mammoplasty	Y	Y	Y	Y
051L	27	Reduction mammoplasty	Y		Y	Y
172L	28	Reduction mammoplasty	Y		Y	Y
C023	35	Prophylactic mastectomy	Y			
C128	59	Prophylactic mastectomy	Y			
153	60	Reduction mammoplasty			Y	Y
112R	61	Reduction mammoplasty	Y		Y	Y
237	66	Reduction mammoplasty	Y	Y	Y	Y

### Immunofluorescence

All samples were fixed with 4% formaldehyde for 20 min and then incubated in blocking buffer (10% heat-inactivated goat serum in PBS + 0.5% Triton X-100) at 4 °C for at least 1 day. Primary antibodies were diluted in blocking buffer. After at least 1 day incubating at 4 °C with the primary antibodies, samples were washed several times with PBS + Triton X-100 for at least one day and incubated with fluorophore-conjugated secondary antibodies diluted at a concentration of 1:200 in blocking buffer for approximately 1 day. All samples were washed with PBS + 1 μg ml^−1^ DAPI for at least 1 h before imaging. Antibodies and stains were used, as summarized in Table 3.

**Table 3:** Antibodies and stains used

Antibody	Product	Application
Anti-human keratin 19	BioLegend 628,502 (clone A53-B/A2)	IF (1:1000)
Anti-human keratin 14	BioLegend 905,301 RB-9020-P (clone Poly19053)	IF (1:1000)
Anti-human CD104	Chemicon MAB1964 (clone 3E1)	IF (1:1000)
Anti-human CD133-PE	BioLegend 372,804 (clone 7)	FC (1:200)
Anti-human CD10	BioLegend 312,210	FC (1:200)
Anti-human CD117-APC	BioLegend 313,206 (clone 104D2)	FC (1:200)
Anti-human CD271-APC	BioLegend 345,108 (clone ME20.4)	FC (1:200)
Anti-human CD227	BD 743,308	FC (1:200)

### Flow cytometry

Each sample was transferred to a collection tube and resuspended in PBS/1% BSA/5mM EDTA. Fluorescently tagged antibodies were added at concentrations presented in Table 3 and incubated for 30 min on ice. Labeled cells were washed three times with PBS to remove unbound antibody and resuspended in flow buffer (PBS with 2% BSA, 1 mM EDTA, and 1 μg/mL DAPI). Cells were sorted on a BD FacsAria III. LEps were defined as CD133+ /CD271− cells, and MEps were defined as CD133−/CD271+ cells, with DAPI + cells discarded. Compared to cells from standard 2-D cultures, postconfluent cells exhibit marked autofluorescence.

### Image acquisition

All confocal microscopy images were acquired using a Zeiss LSM 880 with Airyscan running Zeiss Zen Software. Subsequent deconvolution was performed with AutoQuant. All bright-field microscopy images were acquired using a Nikon Eclipse Ti-E with stage-top incubation and high-speed electromagnetic stage with piezo Z, running Nikon Elements software. Subsequent workup and image analysis was performed using ImageJ.

### Mechano-NPS analysis

Mechanical phenotyping was done as previously described [20]. Briefly, microfluidic channels were fabricated from PDMS atop a glass substrate with Pt electrodes and Au contact pads. Channels were 30 μm high, with a 2055-μm-long by 10–12-μm-wide contraction channel, flanked on either side by 85-μm nodes and 25-μm pores. Cells were trypsinized to monodispersity before being flowed through the microfluidic channel with a non-pulsatile pressure of ~ 21 kPa with a constant voltage of 1 V across the channel. Cytoskeleton-altering drugs, such as latrunculin, were not used for these experiments. The wCDI index was calculated as previously described [20] with a correction made for velocity.

### RNA-seq

Total RNA from FACS-sorted cells was isolated using Quick DNA/RNA Microprep Plus Kit (Zymo Research). RNA library preparation was done with either the KAPA mRNA Hyper kit (cat# KK8581) or the Takara SMART-Seq v4 Ultra Low Input RNA kit (cat# 634,888). Sequencing was done on an Illumina HiSeq 2500 by the City of Hope Integrative Genomics Core Facility. Reads were aligned to *Homo sapiens* reference genome hg19 using TopHat2. All relevant codes to reproduce the analysis are available in Additional file 1: Code 1. PANTHER inquiries were submitted to http://www.pantherdb.org.

### Statistics

For mechanical analysis in Fig. 4, two-way ANOVA was performed with wCDI as the dependent variable, specimen and wCDI as the independent variables, *p* values determined by *F* test, and post hoc analysis done with Tukey’s test. For viability and senescence analysis in Additional file 2: Fig. S1A and Additional file 3: Fig. S3A, a repeated-measures linear model was used as per the model *viability/senescence* ~ *timepoint* + *(1 | specimen)*, treating timepoint as a fixed effect and specimen as a random effect (data in Additional file 4: Data 1). Timepoint was modeled as a categorical predictor instead of a continuous predictor for simplicity’s sake, given its expected nonlinear sigmoidal effect. Viability and senescence measurements were logit-transformed to satisfy the normality assumption of linear regression. Estimated marginal mean contrasts were used for the post hoc test, when appropriate. For PCNA analysis in Additional file 2: Fig. S1B, a two-way ANOVA was used as per the model *PCNA* ~ *culture_type* + *cell_type* (data in Additional file 5: Data 2). Tukey’s HSD was used as the post hoc test. All *p* values less than 0.001 were reported as *p* < 0.001.

## Results

### Human mammary epithelial cells develop multilayered morphology under postconfluent conditions

**Fig. 1:**
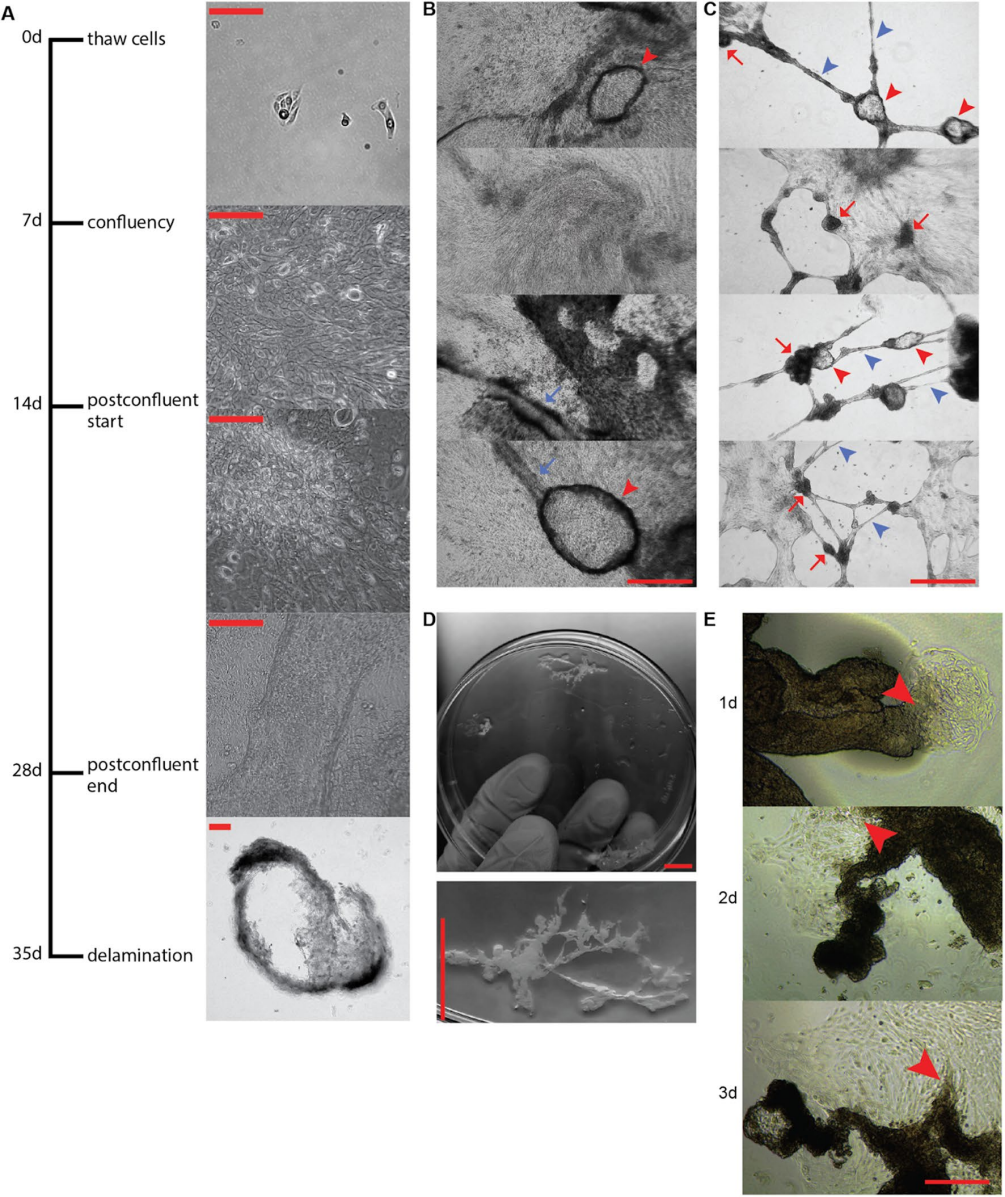
Human mammary epithelial cells develop multilayered morphology under postconfluent conditions. **A** Timeline. Characteristic microscopy for each of the named stages at right. Scale bars are 100 μm. **B** Postconfluent cultures yield dome-like structures (red arrowheads) and tubelike structures (blue arrows). Scale bar is 250 μm. **C** Postconfluent cultures grown on low-stiffness substrates yield lucent spheroids (red arrowheads), dark spheroids (red arrows), and cable-like structures (blue arrowheads). The surrounding regions are sometimes cleared of cells. Scale bar is 250 μm. **D** Collagenase treatment causes postconfluent cultures to condense into branching structures reminiscent of mammary organoids. Scale bars are 1 cm. **E** In intimate contact with plastic, these branching structures show i) focal attachment, ii), enlargement of attached regions, and iii) cellular proliferation. Scale bar is 250 μm

Under standard culture conditions, normal pre-stasis HMECs are grown from frozen ampoules at a starting density of 32 cells/mm^2^ to 70–85% confluency across a period of 5–7 days (Fig. 1A). Culturing HMECs past confluency inhibits cellular proliferation pursuant to trypsinization, and consequently HMECs are typically kept at subconfluent density to permit continued proliferation. However, as long as cultures are refed every-other-day and not trypsinized, postconfluent cells gradually proliferate into a multilayered culture. As measured by ethidium homodimer, the viability of postconfluent culture stays around 99% for up to 4 weeks, whereupon it begins to decline (Additional file 2: Fig. S1A). As measured by PCNA expression, proliferation significantly decreases, with an apparent magnitude of about fivefold, within 3 weeks of culture (Additional file 2: Fig. S1B). Postconfluent cultures were maintained for up to 4 weeks, before cells began to irreversibly delaminate from the culture dish to form macroscopic, floating flakes.

The morphology of postconfluent culture varies with the culture substrate. We have previously characterized the behavior of HMECs on low-stiffness collagen-coated polyacrylamide gels [7] and chose to evaluate postconfluent culture on the same substrates. On tissue culture plastic, postconfluent culture is characterized by a multilayered culture (Fig. 1B). On 2 kilopascal collagen-coated polyacrylamide, this multilayered postconfluent culture progresses to a spheroids-and-cables morphology (Fig. 1C), in which substantial portions of the culture form floating macroscopic structures that are distally tethered to the substrate. The “cables” in these structures can extend several millimeters (Additional file 2: Fig. S1C).

Overnight collagenase digestion of postconfluent cultures on tissue culture plastic causes compaction of cells into free-floating structures with gross morphology reminiscent of epithelial organoids obtained from freshly dissected breast tissue (Fig. 1D). Pursuant to prolonged contact with tissue culture plastic, these pseudo-organoids form adhesive interfaces, which gradually expand and lead to the proliferation of cells on the plastic (Fig. 1E), and these outgrowths can persist without delamination for at least 19 days (Additional file 2: Fig. S1D). This pseudo-organoid intermediate is the only method we have identified that is capable of facilitating the proliferation of HMECs that have entered postconfluent culture, and its morphology is reminiscent of epithelial outgrowths from primary organoids on tissue culture plastic, as we previously described for establishing pre-stasis HMEC strains [21].

### Postconfluent cultures are enriched for luminal cells

**Fig. 2:**
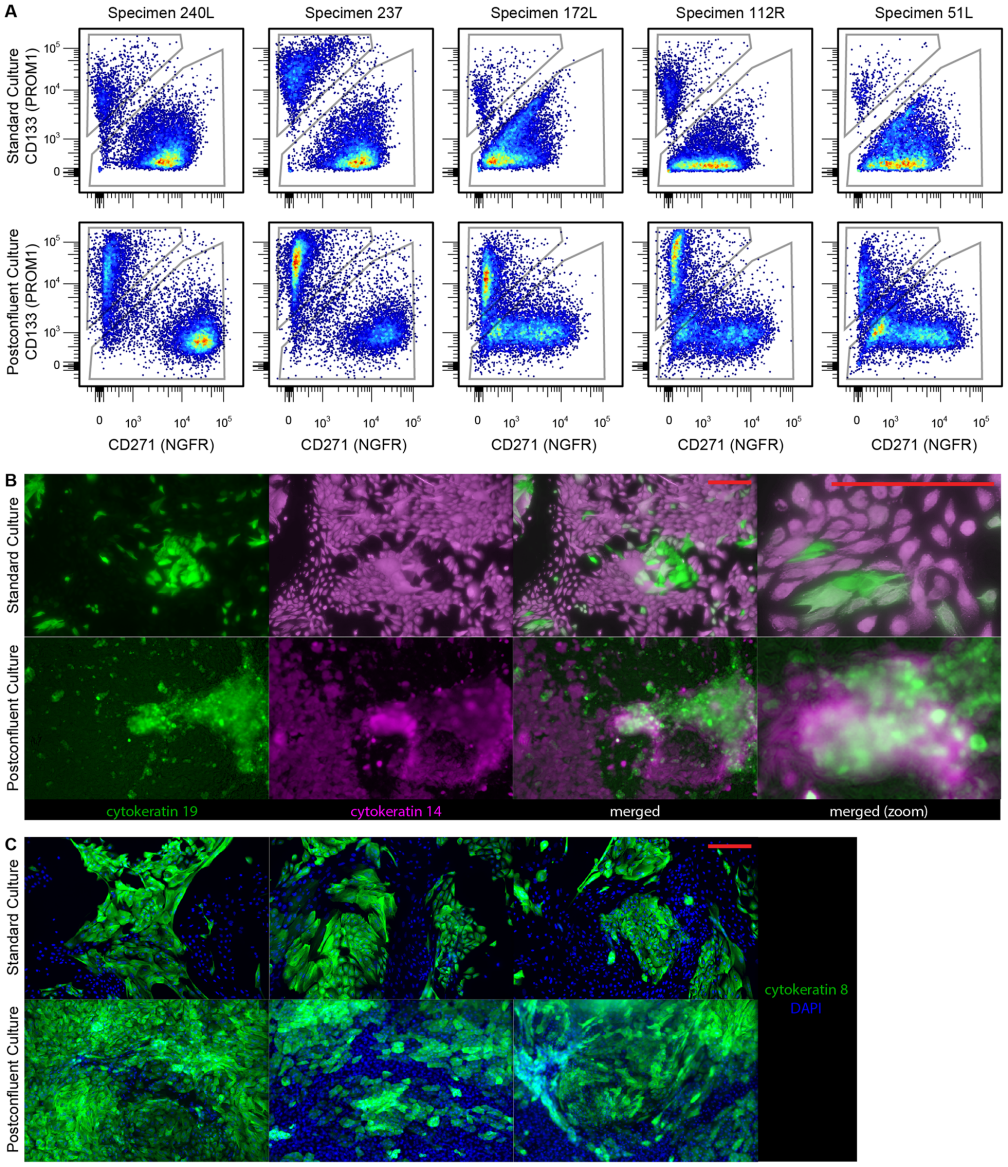
Postconfluent cultures are highly enriched for luminal cells. **A** Flow cytometry using CD133 to identify LEps and CD271 to identify MEps shows enriched abundance of LEps in postconfluent culture as compared to specimen-matched standard culture. Quantified lineage compositions for each of these flow plots are given in Table 4. *n* = 5 distinct HMEC specimens were evaluated. **B** Immunostaining using KRT19 to identify LEps and KRT14 to identify MEps shows enriched abundance of LEps in postconfluent culture as compared to specimen-matched standard culture. **C** Immunostaining using KRT8 to identify LEps. Scale bars are 100 μm

With prolonged HMEC culture that requires passaging on tissue culture plastic, the fraction of LEps gradually drops, becoming a vanishingly small minority of the mixed cell population by the 9th or 10th passage [5]. Postconfluent culture appears to reverse this trend. We performed flow cytometry, defining LEps as CD133+ /CD271− and MEps as CD133−/CD271+ . Flow cytometry for LEps and MEps shows an elevated proportion of CD133+ /CD271− LEps across multiple HMEC specimens in comparison with specimen-matched standard 2D culture (Fig. 2A) (Table 4). Immunostaining postconfluent cultures for LEp marker cytokeratin 8 and 19 and MEp marker cytokeratin 14 confirms this result, showing enrichment of cells bearing luminal cytokeratins (Fig. 2B, C).

**Table 4:** Lineage composition of standard 2D and postconfluent culture (as percent of DAPI-negative cells)

Specimen	Standard culture		Postconfluent culture	
	% LEP	% MEP	% LEP	% MEP
240L	11	81	32	60
273	25	74	32	35
172L	5	82	32	35
112R	9	77	49	32
051L	4	92	22	44

### Postconfluent culture enriches expression of lineage markers and extracellular matrix genes

RNA-seq (Additional files 6 and 7: Data 3 and 4) was performed on MEps and LEps across several specimens from primary epithelial organoids, postconfluent culture, and our standard subconfluent culture (see Methods). Cells were lineage-separated by FACS prior to RNA-seq, with LEps defined as CD133+ /CD271− and MEps defined as CD133−/CD271+ . Principal component analysis showed that the overall gene expression profile of HMECs was segregated by culture method (Additional file 8: Fig. S2A), and we sought to determine what specific changes in gene expression distinguished these three groups.

**Fig. 3:**
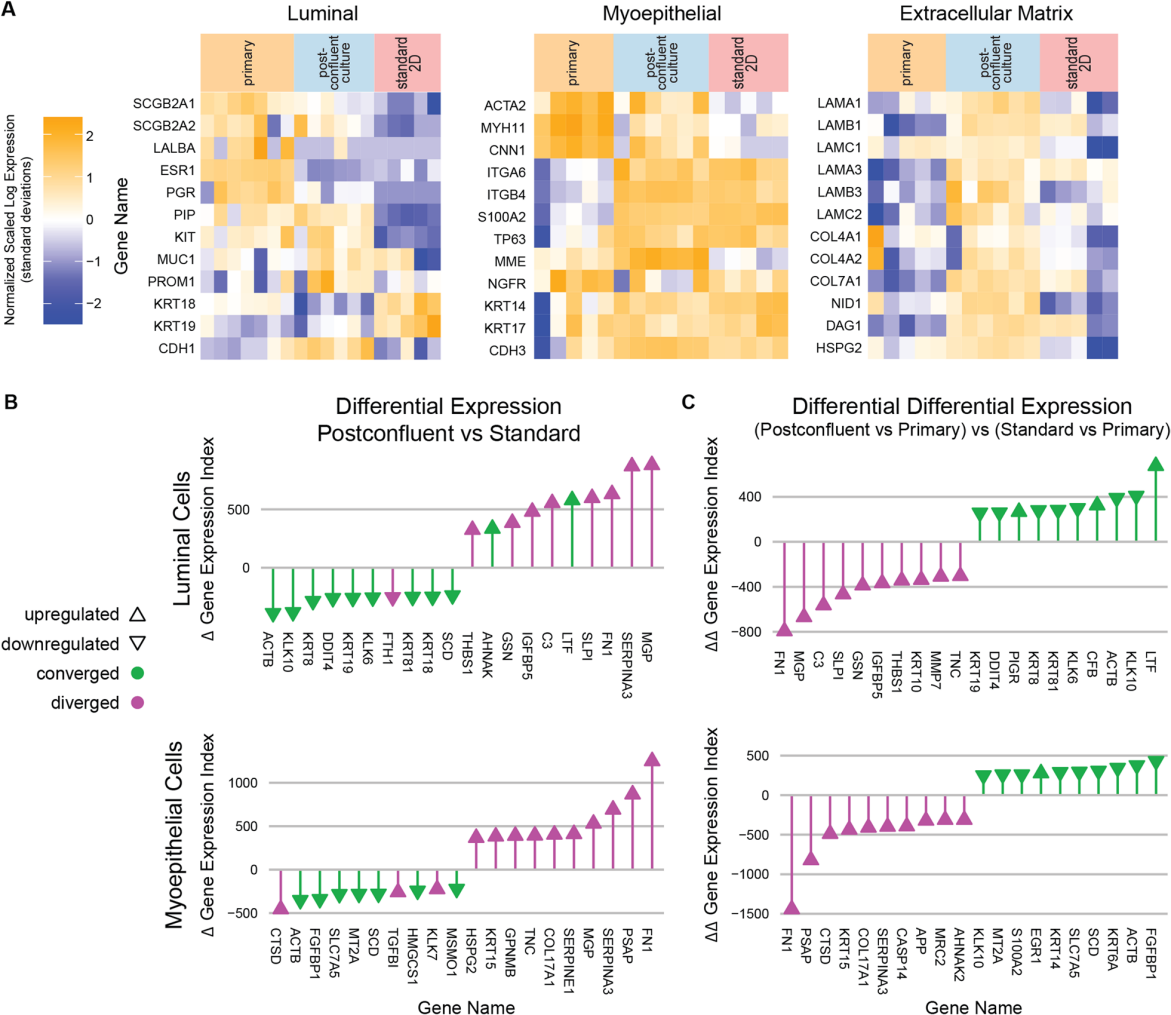
Gene expression of postconfluent culture is intermediate between regular culture and primary tissue. **A** Relative gene expression for characteristic genes of luminal cells, myoepithelial cells, and the extracellular matrix genes for standard two-dimensional culture, postconfluent culture, and primary tissue specimens. **B** Lollipop plot showing the genes with the greatest differential expression index between postconfluent culture and standard 2D culture. **C** Lollipop plot showing the genes with the greatest convergence and divergence of differential expression index between postconfluent culture and primary tissue specimens. The differential expression index, as described in the text, is defined in Additional file 8: Fig. S2B

Gene expression was evaluated for a predetermined set of lineage markers for LEps and MEps [22] as well as for extracellular matrix components (Fig. 3A).

Postconfluent LEps share more lineage markers with primary organoids than subconfluent LEps. First, postconfluent LEps express the mammary epithelial tissue-restricted markers mammaglobins A and B (*SCGB2A1* and *SCGB2A2*) [23] at a level similar to primary tissue samples. Second, postconfluent LEps express the apocrine epithelial marker prolactin-inducible protein *PIP* (aka *GCDFP-15*) [24] at a level similar to primary tissue samples. Third, postconfluent LEps express the putative progenitor marker *KIT[5]*at a level similar to primary tissue [5], but further results, shown below, suggest this is due to a distinct subset of c-Kit+ luminal cells. These results suggest that postconfluent LEps may have greater differentiation than subconfluent LEps. Luminal cytokeratins *KRT18* and *KRT19* show reduced gene expression in postconfluent culture relative to standard culture, but there is a poor correlation between gene expression and protein levels for cytokeratins [25, 26], making interpretation of this finding fraught. Expression of the hormone receptors *ESR1* and *PGR* is no higher in postconfluent culture than it is in standard culture; the negligible expression of hormone receptors in cultured HMECs is a persistent problem in the field that limits experiments involving estrogen or progesterone, and postconfluent culture does not change this.

Postconfluent MEps show elevated expression of contractility-associated lineage markers. Specifically, postconfluent MEps express *ACTA2*, *MYH11*, and *CNN1* at levels greater than subconfluent culture but less than primary organoids. These results suggest that postconfluent MEps may have greater differentiation than subconfluent MEps.

Postconfluent cells show elevated expression of genes encoding structural components of the basement membrane. Gene expression was summed between specimen-matched MEps and LEps when analyzing basement membrane genes. Compared to subconfluent cells, postconfluent cells have higher expression for genes for laminin-111 (*LAMA1, LAMB1, LAMC1*), laminin-332 (*LAMA3, LAMB3, LAMC2*), collagen IV (*COL4A1, COL4A2*), and accessory proteins (*NID1, DAG1, HSPG2*). These results suggest that postconfluent cells produce more basement membrane than subconfluent cells.

To identify the genes that distinguished primary epithelial organoids, postconfluent culture, and our standard subconfluent culture, we sorted genes with a differential expression index, calculated as the geometric mean of ratio-based differential expression (i.e., DESeq2 analysis) and difference-based differential expression (Additional file 8: Fig. S2B). Using this index, we identified both the genes with the greatest differential expression between postconfluent culture and standard 2D culture (Fig. 3B) as well as the genes in postconfluent culture that converged or diverged the most from primary samples relative to standard 2D culture (Fig. 3C). For both LEps and MEps, matrix Gla protein (*MGP*), serpin A3 (*SERPINA3*), and fibronectin (*FN1*) are upregulated versus standard culture, but this upregulation constitutes a divergence from primary samples. For LEps specifically, lactotransferrin (*LTF*) stands out as an upregulated gene that constitutes a convergence toward primary organoid samples. For MEps, prosaposin (*PSAP*) stands out as an upregulated gene whose upregulation constitutes a divergence from primary samples.

To combine gene expression changes into gene network changes, we analyzed the 200 most converged and 200 most diverged genes (200 being approximately 1% of the genome features in this dataset) with PANTHER [27] using the GO-Slim Molecular Function pathways. The statistically significant (false discovery rate-adjusted *p* < 0.05) gene sets and fold enrichments are given in Table 5, and the breakdown of specific enrichments is shown in Additional file 9: Data 5. For LEps, no pathways were significantly enriched among convergent genes. For MEps, several sets of pathways appear among convergent genes, including mitochondrial pathways, cytoskeletal pathways, and protein localization pathways. For both MEps and LEps, various extracellular matrix and extracellular matrix-regulating pathways were among divergent genes.

**Table 5:** Gene set enrichment analysis for postconfluent culture

Convergent gene expression		Divergent gene expression	
Gene set	Fold enrichment	Gene set	Fold enrichment
*Myoepithelial cells*			
Mitochondrial translational elongation^a^	11	Collagen binding	22
Mitochondrial translational termination^a^	10	Extracellular matrix structural constituent	12
Protein localization to plasma membrane	6	Integrin binding	10
Mitochondrial transport	4	Calcium ion binding	7
Supramolecular fiber organization	3	Metalloendopeptidase activity	6
Chromosome organization	3		
Cellular component biogenesis	2		
*Luminal cells*			
		Integrin binding	16
		Extracellular matrix structural constituent	16
		Serine-type endopeptidase inhibitor activity^b^	8
		Protease binding	7
		Metalloendopeptidase activity^b^	7
		Serine-type peptidase activity^b^	5
		Calcium ion binding	4

^a^the majority of matched genes in these sets overlap

^b^the majority of matched genes in these sets overlap

### Senescent cells accumulate in postconfluent cultures

Senescence, as measured by markers such as senescence-associated β-galactosidase (SAβG) activity and p16 expression, becomes increasingly prevalent with long-term HMEC culture [19]. Although these determinations are usually made with serially passaged HMECs, it would be reasonable to assume that senescence similarly increases in postconfluent culture. We assayed for SAβG activity in postconfluent cultures of varied duration and measured the corresponding SAβG + area fraction (Additional file 3: Fig. S3A). We observed a SAβG + area fraction starting at ~ 5% for confluent culture, rising to ~ 18%, ~ 30%, and ~ 35% SAβG for 1wk, 2wk, and 3wk postconfluent culture, respectively. Next, gene expression was evaluated for a predetermined set of senescence markers (Additional file 3: Fig. S3B), which showed elevated postconfluent expression of *CDKN2A*/*CDKN1A*/*TP53* (p16/p21/p53). We did not see elevated postconfluent expression of senescence-associated apoptosis-regulating *BCL* genes. Among senescence-associated secretory phenotype genes, interleukins (*IL1A/IL6*) were not elevated, but extracellular metalloproteases (*MMP1/MMP13*) were elevated. In total, the evidence is consistent with a fraction of postconfluent cells becoming senescent.

### Postconfluent culture cells are less deformable than cells in standard 2D cell culture

Cellular mechanical properties are a function of the structure and dynamics of the cytoskeleton, cell membrane, nucleus, and extracellular matrix, and, correspondingly, cellular states such as differentiation [28], malignancy [29], and aging [30] often correlate with mechanical properties. Considering the increased ECM expression of postconfluent cells and their dense, multilayered microenvironment, we hypothesized that mechanical properties might vary between postconfluent cultures and standard 2D cultures.

**Fig. 4:**
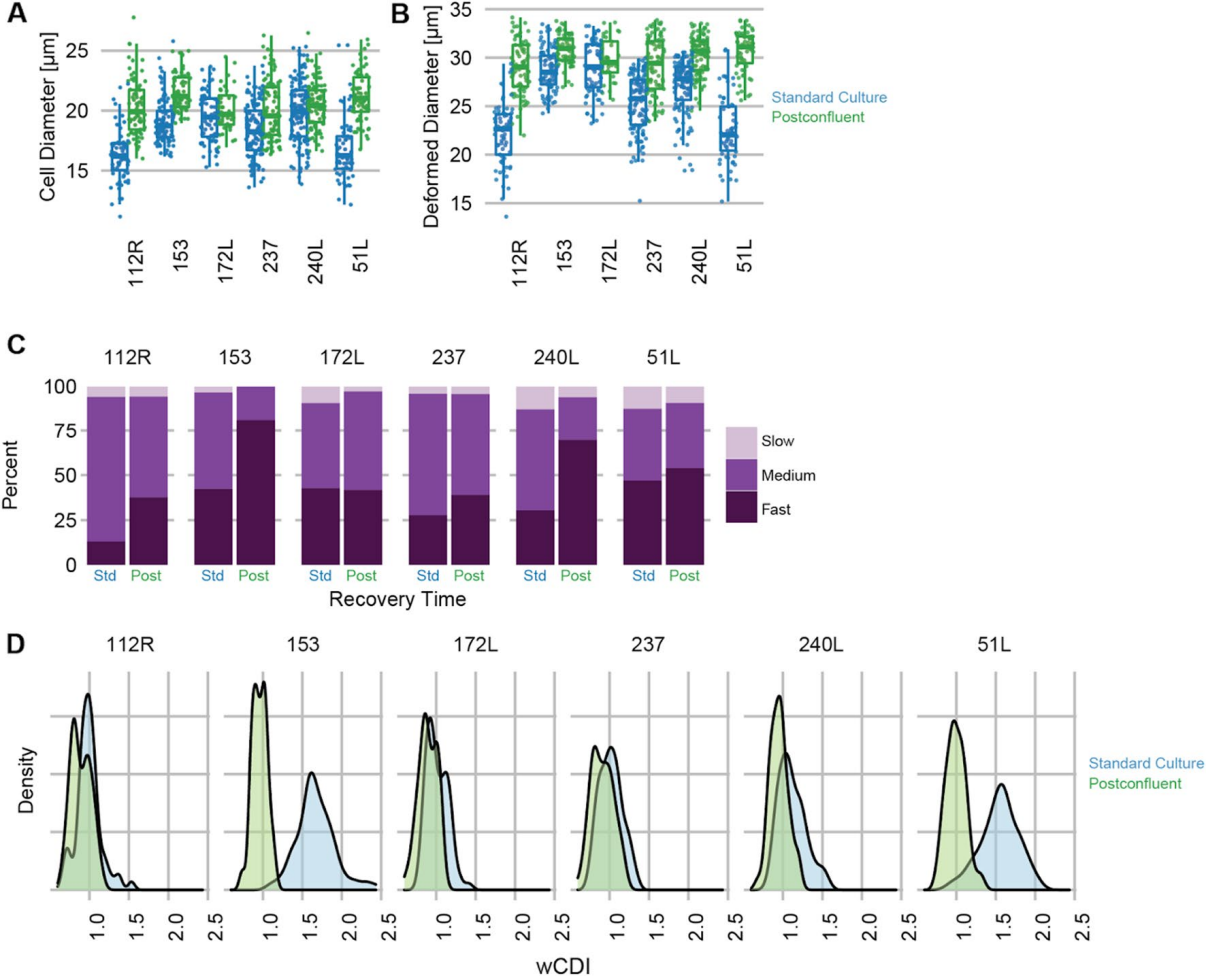
Postconfluent culture cells have reduced deformability. Cells from postconfluent culture had their physical properties compared to cells from regular culture using mechano-node pore sensing (mechano-NPS), showing **A** relaxed cell diameter, **B** deformed cell diameter, **C** recovery time, and **D** the composite whole-cell deformability index. *n* = 6 distinct HMEC specimens were evaluated for mechanical analysis

Using mechano-node-pore sensing (mNPS) [20], we assessed the physical properties of postconfluent cells versus specimen-matched controls from standard 2D cell culture at the single-cell level. Using mNPS, across six specimens, we assessed cell diameter (Fig. 4A), deformed cell diameter (Fig. 4B), and cell recovery time after transverse deformation (Fig. 4C), between cells from standard culture and cells from postconfluent culture for each specimen. Next, we calculated the whole-cell deformability index (wCDI), which combines these measurements, as a composite metric of mechanical phenotype (Fig. 4D). We used two-way ANOVA to assess the significance of postconfluent culture and specimen identity on wCDI (Table 6), which showed an effect that was significant (*F* test *p* value < 0.001) and large (*η*^2^ = 0.23, Cohen’s *F* = 0.55). A post hoc Tukey’s test showed that postconfluent culture was associated with a significant drop of wCDI (i.e., deformability) of varied magnitude across the six tested specimens. We consider the postconfluence-associated decrease in HMEC wCDI interesting in the context of the cancer-and-immortalization-associated increase in HMEC wCDI [20].

**Table 6:** Mechano-NPS ANOVA statistics

Effect	*p* value	*η*^2^	Cohen’s *F*
Culture type	< 2e−16	0.23	0.55
Specimen	< 2e−16	0.31	0.68
Culture type:specimen	< 2e−16	0.17	0.45

Contrast (Tukey’s HSD)	*p* value	Δ*µ*
112R normal vs postconf:112R	< 0.014	0.1
153 normal vs postconf:153	< 1e–7	0.71
172L normal vs postconf:172L	< 0.011	0.12
237 normal vs postconf:237	< 1e–4	0.11
240L normal vs postconf:240L	< 1e–7	0.17
51L normal vs postconf:51L	< 1e–7	0.55

### Postconfluent cultures are enriched for c-Kit+ cells

**Fig. 5:**
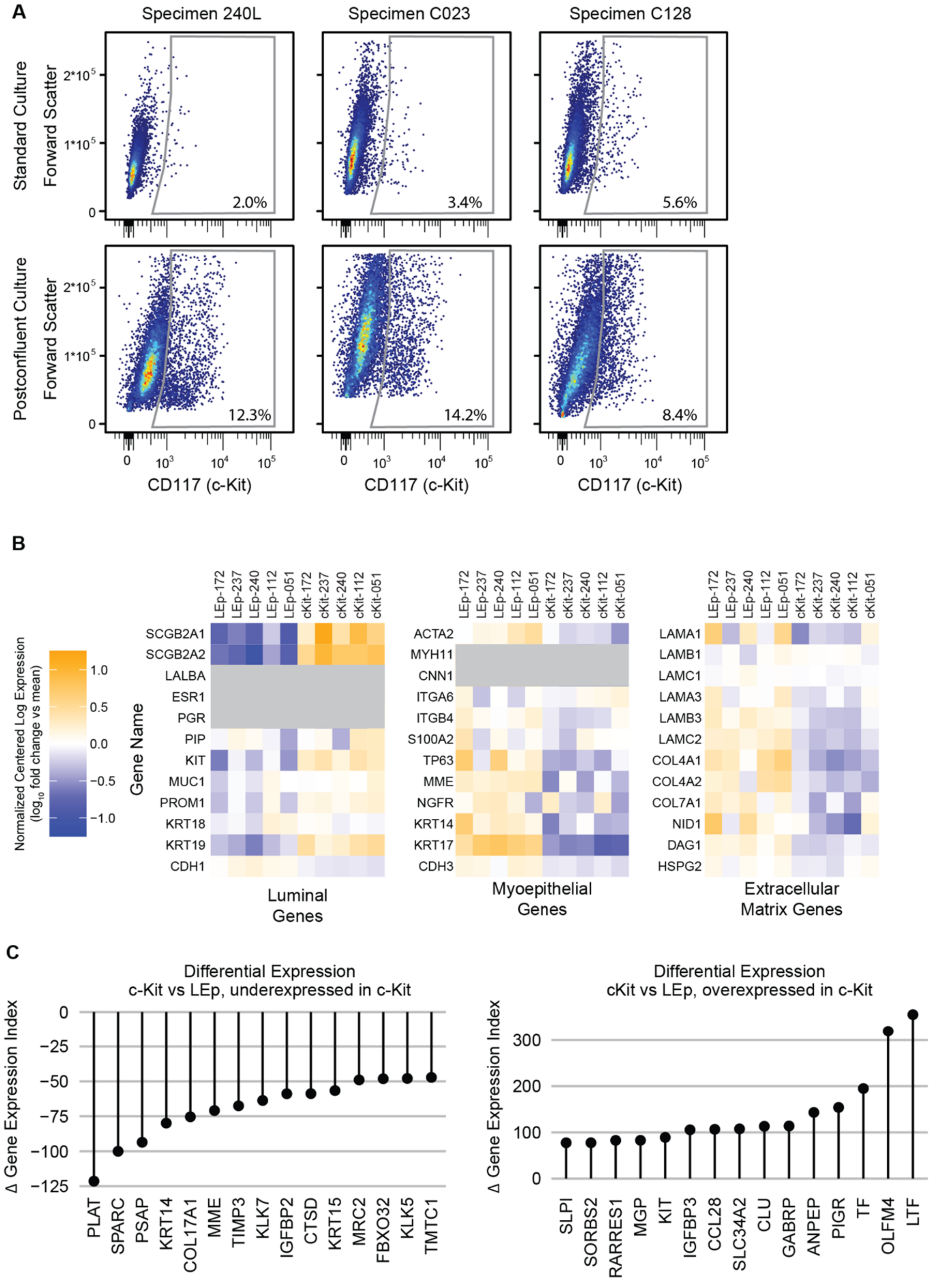
Postconfluent cultures are enriched for c-Kit+ cells. **A** Flow cytometry report showing the c-Kit+ subpopulation. **B** Relative gene expression for characteristic genes of luminal cells, myoepithelial cells, and the extracellular matrix genes for LEp (CD133+) and c-Kit+ subpopulations of HMECs from five specimens. Genes of interest with RNA reads fewer than 2 CPM across all specimens are blocked out in gray. **C** Lollipop plot showing the genes with the greatest differential expression index between LEps and c-Kit+ cells. The differential expression index, as described in the text, is defined in Additional file 8: Fig. S2B. *n* = 6 distinct HMEC specimens were evaluated for c-Kit analysis

We examined whether the composition of cells in postconfluent culture varied aside from enrichment for LEps. Flow cytometry was used to measure the fraction of cells expressing c-Kit, a marker previously demonstrated to exhibit human mammary epithelial progenitor activity [22]. Postconfluent cells show a markedly enriched c-Kit+ population in comparison with standard 2D culture across several HMEC specimens (Fig. 5A).

To characterize the properties of postconfluent c-Kit+ cells, we performed RNA-seq on postconfluent c-Kit+ cells across five specimens. Cells were separated by FACS prior to RNA-seq, with c-Kit+ cells defined as CD117+ , LEps defined as CD133+ /CD271−, and MEps defined as CD133−/CD271+ . In comparison with LEps, specimen-matched c-Kit+ cells show increased expression of most canonical luminal gene markers—*KIT* itself, but especially *SCGB2A1* and *SCGB2A2* (Fig. 5B). c-Kit+ cells also show decreased expression of most canonical myoepithelial gene markers and extracellular matrix proteins. It is unclear whether this effect is due to differential MEp contamination between these groups or whether there is a “super-luminal” quality to the c-Kit+ cells.

Whole-genome geometric mean index analysis of c-Kit+ cells showed strong overexpression of several interesting genes (Fig. 5C). In particular, several genes involved in LEp mucosal immunity—lactoferrin (LTF) [31], polymeric immunoglobulin receptor (*PIGR*) [32], and olfactomedin 4 (*OLFM4*) [33] were overexpressed. Other hits include latexin-like (*RARRES1*), which regulates stem cell survival [34], matrix Gla protein (*MGP*), which may be a stem cell niche maintenance factor [35], and aminopeptidase N (*ANPEP*, aka CD13), which is an ectoenzyme metalloprotease that is a putative LEp surface marker [36].

Pathway analysis with PANTHER GO-slim Molecular Functions shows results that would not have been predicted from the geometric mean index analysis alone (Additional file 10: Data 6) (Table 7). Phosphatidylinositol 3-kinase binding and protein kinase activity pathways are upregulated in postconfluent c-Kit+ cells. Seine proteases, calcium-binding proteins, and cell adhesion molecules are downregulated in postconfluent c-Kit+ cells.

**Table 7:** Gene set enrichment analysis for c-Kit+ cells in postconfluent culture

Upregulated gene expression		Downregulated gene expression	
Gene set	Fold enrichment	Gene set	Fold enrichment
*c-Kit+ cells*			
Phosphatidylinositol 3-kinase binding	17	Serine-type endopeptidase inhibitor activity^a^	9
Protein kinase activity	3	Calcium ion binding	7
		Serine-type endopeptidase activity^a^	6
		Cell adhesion molecule binding	5

^a^the majority of matched genes in these sets overlap

## Discussion

Postconfluent culture enriches the CD133+ /CD271− LEp and c-Kit+ subpopulations of HMEC culture, which is useful because these subpopulations tend to be under-represented in standard 2-D cultures [5]. In some cases, postconfluent culture yields LEp and c-Kit+ subpopulations when none would be present in standard culture. Primary mammary tissue specimens typically have a composition rich in LEps [2], and based on our findings we speculate that postconfluent culture nudges the lineage composition of cultured HMECs closer to the primary-like state. Although HMECs can be cultured from a wide variety of mammoplasty and mastectomy specimens, not all specimens yield isolable subpopulations of LEp or c-Kit+ cells under standard culture conditions. Postconfluent culture lets more subpopulations be propagated from such specimens. Considering the centrality of LEps and c-Kit+ cells to breast cancer research [37, 38], we consider this subpopulation expansion to be invaluable.

Postconfluent culture alters HMEC gene expression, in some respects, to be more like that of primary specimens. Specifically, postconfluent HMECs show more highly differentiated gene expression, such as the upregulation of lineage-specific mammaglobins and prolactin-inducible protein in LEps and lineage-specific smooth muscle actin, myosin, and calponin in MEps. The increased expression of essentially all basement membrane genes, in combination with a more equal ratio of LEps to MEps in a multilayered culture, suggests that the postconfluent microenvironment may be more similar to the physiological microenvironment. Although organoid culture is still the gold standard for eliciting primary-like gene expression from HMECs [39], postconfluent culture has the advantage of not requiring three-dimensional culture techniques, simplifying establishing cultures and recovering cells. As such, postconfluent culture is a useful compromise between standard culture and organoid culture.

The decreased deformability of postconfluent cells is perhaps the most distinct physical feature of these cultures, after their multilayered nature. A plausible, but currently unsubstantiated, hypothesis is that elevated and sustained ECM expression causes cellular deformability to drop, considering that ECM proteins such as collagen are stiff and that only so much of these proteins can accumulate in standard cultures. An important caveat to this analysis is that it has been done with unsorted mixtures of LEps and MEps, which have differing mechanical properties, but our statistical model suggests that altered lineage composition is insufficient to explain the decreased postconfluent deformability.

The growth characteristics of postconfluent culture are noteworthy. In standard HMEC culture, normal LEps grown in isolation, such as FACS-sorted LEps, do not grow well, and in standard 2-D mixed lineage cultures luminal cells do not last much beyond 9th or 10th passage [5]. Although expansion of purified luminal cells has been reported, purified luminal cells tend to convert to myoepithelial cells, as reported in the literature [6] and in our own unpublished work. We have speculated that the reason we have been more successful than most at maintaining primary normal luminal cells in culture is that the milieu of cell types present generate a growth-conducive microenvironment. Even in 2-D cultures, we observe that luminal cells grow as islands surrounded by fences of myoepithelial cells, and careful inspection of the interfaces reveals that myoepithelial cells protrude beneath the luminal cells for a distance. We think that postconfluent cultures work to produce such an abundance of luminal cells because the different lineages are recreating the ECM and cellular microenvironment that is ideal for their growth.

Although contact inhibition typically prevents epithelial cell culture from growing past confluency, postconfluent HMECs can continue proliferating for several weeks past the expected point of contact inhibition. Low-stress media, such as M87A [4], appears to be necessary for maintaining postconfluent growth. Although M87A reduces the accumulation of senescent cells when compared to other culture media, our data indicates that postconfluent cells eventually become senescent all the same. Determining how to further delay the onset of senescence is important for cell culture in general and postconfluent culture in particular. During the course of the experiments for this paper, a SARS-COV2 pandemic-related supply chain issue temporarily introduced oxidized tyrosine, a known cause of oxidative stress [40], into our culture media. During this period, none of our HMEC specimens could enter postconfluent culture, instead growth arresting upon confluence. Further investigation would be valuable into what growth media is compatible with postconfluent culture.

Although postconfluent culture becomes non-viable within 35 days due to cellular delamination, it is notable that enzymatic processing allows postconfluent cells to form pseudo-organoids, which are competent to re-attach to plastic and grow out. As a future direction, it may be possible to serially passage postconfluent HMECs through this pseudo-organoid intermediate.

The lessons learned in HMEC culture may be broadly applicable to other cell cultures. Historically, HMEC culture used to rely on conditioned media [41] until defined media were formulated that minimized this need [42]. The defined media were optimized for clonal growth rather than ensemble growth. The same trend has been seen in other culture systems, such as prostate culture that once required fibroblast feeder layers [43], but now equivalent media additives have come to be used [44]. Similarly, Engelbreth-Holm-Swarm gel-based three-dimensional culture was popularized starting in 1987 by Mina Bissell for mammary culture [45], and it has since become one of the most widely used substrates for organoid culture [46]. In the same vein, we hope that postconfluent culture of HMECs may be the forerunner to many further postconfluent cultures. We speculate that low-stress media that are optimized for ensemble growth will be the key to establishing postconfluent cultures in other primary cell contexts.

## Conclusions

In conclusion, postconfluent culture is a novel, useful strategy for altering the lineage composition of HMECs, by increasing the proportion of luminal and progenitor cells. Postconfluent culture creates a microenvironment with cellular composition closer to the physiological state and eases the isolation of scarce cell subtypes. As such, postconfluent culture is a valuable tool for researchers using HMECs for breast cancer research.

## Supplementary Information

**Additional file 1**: **Code 1**. Analysis pipeline. Related to Figs. 3 and 5. R code sufficient to recapitulate all analysis in Figs. 3 and 5, as well as statistics used in all other figures.

**Additional file 2**: **Fig. S1**. **A** Viability of postconfluent cultures over time, as measured by ethidium homodimer incorporation. Each color indicates an independent HMEC specimen (*n* = 6). Estimated marginal mean post hoc analysis for a repeated measures linear model of logit-transformed percent viability (see Methods) confirms that the viability of postconfluent cultures does not significantly drop until week four, with *p* < 0.001. **B** Proliferation of postconfluent cultures, as measured by proliferating cell nuclear antigen (PCNA). Tukey’s HSD post hoc analysis confirms that counts-per-million PCNA gene expression significantly drops from confluent to postconfluent culture (*p* < 0.001), but postconfluent expression is not significantly different from primary expression. **C** Macroscopic structure from postconfluent HMEC culture on 2 kPa collagen-coated polyacrylamide. Attachment points to substrate denoted with red arrowheads. **D** Advanced time points for postconfluent structures detached from plastic with collagenase, then reattached and grown out on fresh dishes. Columns are replicates. The black regions visible in the 19d time point are fiducial ink markings drawn on the underside of the dish. Scale bars are 1 mm.

**Additional file 3**: **Fig. S3**. **A** Senescence of postconfluent cultures over time, as measured by area fraction of cells senescence-associated β-galactosidase activity (SABG). Each color indicates an independent HMEC specimen (*n* = 6). Estimated marginal mean post hoc analysis for a repeated measures linear model of logit-transformed percent SABG positive cells (the Methods) confirms that the senescence of postconfluent cultures rises over time, with *p* < 0.001. **B** Relative gene expression for characteristic senescence-associated genes of FACS-sorted LEps and MEps from three-week postconfluent culture.

**Additional file 4**: **Data 1**. Viability data, Related to Fig. S1A. Contains ethidium homodimer dead cell counts at a sequence of time points.

**Additional file 5**: **Data 2**. Senescence-associated beta-galactosidase activity, Related to Fig. S2A. Contains threshold-based area measurements for SABG + portions of microscopy fields.

**Additional file 6**: **Data 3**. RNA-seq counts, Related to Figs. 3 and 5. Contains the raw counts used for all RNA analysis in these figures. Reads were aligned to Homo sapiens reference genome hg19 and assigned to HGNC symbols. These data are suitable as input for Additional Code 1.

**Additional file 7**: **Data 4**. RNA-seq metadata, Related to Figs. 3 and 5. Contains specimen ID, chronological age, lineage (MEp/LEp), culture type, and library type for the 37 samples analyzed in this figure. These data are suitable as input for Additional Code 1.

**Additional file 8**: **Fig. S2**. **A** Principal component analysis of culture conditions, divided by lineage. **B** Equation for differential expression index.

**Additional file 9**: **Data 5**. Gene ontology analysis, Related to Figure 3. Contains details of gene ontology analysis. All gene hits considered converged or diverged from primary tissue are listed here, as per the methodology described in the Methods. The specific genes found in each of the putative gene sets as well as the full report from geneontology.org.

**Additional file 10**: **Data 6**. Gene ontology analysis, Related to Figure 5. Contains details of gene ontology analysis. All gene hits considered converged or diverged from primary tissue are listed here, as per the methodology described in the Methods. The specific genes found in each of the putative gene sets as well as the full report from geneontology.org.

## Data Availability

The authors assert that all data supporting the findings of this study are available within the paper and its supplementary information files. HMEC strains are made available either by contacting the corresponding author at City of Hope, or by contacting Dr. Martha Stampfer at http://hmec.lbl.gov.

## Abbreviations

HMEC: Human mammary epithelial cell
LEp: Luminal cell
MEp: Myoepithelial cell

## References

[1] Stampfer MR, Garbe JC. Human Mammary Epithelial Cell (HMEC) Bank [Internet]. [cited 2020 Jun 25]. Available from: https://hmec.lbl.gov/

[2] Villadsen R, Fridriksdottir AJ, Rønnov-Jessen L, Gudjonsson T, Rank F, LaBarge MA (2007). Evidence for a stem cell hierarchy in the adult human breast. J Cell Biol.

[3] Inman JL, Robertson C, Mott JD, Bissell MJ (2015). Mammary gland development: cell fate specification, stem cells and the microenvironment. Development.

[4] Lee JK, Bloom J, Zubeldia-Plazaola A, Garbe JC, Stampfer MR, LaBarge MA (2018). Different culture media modulate growth, heterogeneity, and senescence in human mammary epithelial cell cultures. PLoS ONE.

[5] Garbe JC, Pepin F, Pelissier FA, Sputova K, Fridriksdottir AJ, Guo DE (2012). Accumulation of multipotent progenitors with a basal differentiation bias during aging of human mammary epithelia. Cancer Res.

[6] Péchoux C, Gudjonsson T, Ronnov-Jessen L, Bissell MJ, Petersen OW (1999). Human mammary luminal epithelial cells contain progenitors to myoepithelial cells. Dev Biol.

[7] Pelissier FA, Garbe JC, Ananthanarayanan B, Miyano M, Lin C, Jokela T (2014). Age-related dysfunction in mechanotransduction impairs differentiation of human mammary epithelial progenitors. Cell Rep.

[8] Petersen OW, Rønnov-Jessen L, Howlett AR, Bissell MJ (1992). Interaction with basement membrane serves to rapidly distinguish growth and differentiation pattern of normal and malignant human breast epithelial cells. Proc Natl Acad Sci U S A.

[9] McClatchey AI, Yap AS (2012). Contact inhibition (of proliferation) redux. Curr Opin Cell Biol.

[10] Kim J-H, Asthagiri AR (2011). Matrix stiffening sensitizes epithelial cells to EGF and enables the loss of contact inhibition of proliferation. J Cell Sci.

[11] Kim J-H, Kushiro K, Graham NA, Asthagiri AR (2009). Tunable interplay between epidermal growth factor and cell–cell contact governs the spatial dynamics of epithelial growth. Proc Natl Acad Sci U S A Natl Acad Sci.

[12] Pino V, Ramsauer VP, Salas P, Carothers Carraway CA, Carraway KL (2006). Membrane mucin Muc4 induces density-dependent changes in ERK activation in mammary epithelial and tumor cells: role in reversal of contact inhibition. J Biol Chem.

[13] Perrais M, Chen X, Perez-Moreno M, Gumbiner BM (2007). E-cadherin homophilic ligation inhibits cell growth and epidermal growth factor receptor signaling independently of other cell interactions. Mol Biol Cell.

[14] Vizirianakis IS, Chen Y-Q, Kantak SS, Tsiftsoglou AS, Kramer RH (2002). Dominant-negative E-cadherin alters adhesion and reverses contact inhibition of growth in breast carcinoma cells. Int J Oncol.

[15] Padrón JM, van der Wilt CL, Smid K, Smitskamp-Wilms E, Backus HH, Pizao PE (2000). The multilayered postconfluent cell culture as a model for drug screening. Crit Rev Oncol Hematol.

[16] Sukocheva OA, Yang Y, Gierthy JF (2009). Estrogen and progesterone interactive effects in postconfluent MCF-7 cell culture. Steroids.

[17] Gierthy JF, LincolnRoth DWKE, Bowser SS, Bennett JA, Bradley L (1991). Estrogen-stimulation of postconfluent cell accumulation and foci formation of human MCF-7 breast cancer cells. J Cell Biochem Wiley.

[18] LaBarge MA, Stampfer MR, Garbe JC. Media for culturing epithelial cells [Internet]. US Patent. 2022 [cited 2022 Oct 18]. Available from: https://patentimages.storage.googleapis.com/1b/a2/66/ac6e8388ab2f5a/US11293008.pdf

[19] Garbe JC, Bhattacharya S, Merchant B, Bassett E, Swisshelm K, Feiler HS (2009). Molecular distinctions between stasis and telomere attrition senescence barriers shown by long-term culture of normal human mammary epithelial cells. Cancer Res.

[20] Kim J, Han S, Lei A, Miyano M, Bloom J, Srivastava V (2018). Characterizing cellular mechanical phenotypes with mechano-node-pore sensing. Microsyst Nanoeng [Internet].

[21] Labarge MA, Garbe JC, Stampfer MR (2013). Processing of human reduction mammoplasty and mastectomy tissues for cell culture. J Vis Exp [Internet].

[22] Sayaman RW, Miyano M, Senapati P, Shalabi S, Zirbes A, Todhunter ME, et al. Epigenetic changes with age primes mammary luminal epithelia for cancer initiation [Internet]. Cold Spring Harbor Laboratory. 2021 [cited 2021 Mar 7]. p. 2021.02.12.430777. Available from: 10.1101/2021.02.12.430777v1

[23] Fleming TP, Watson MA (2000). Mammaglobin, a breast-specific gene, and its utility as a marker for breast cancer. Ann N Y Acad Sci.

[24] Viacava P, Naccarato AG, Bevilacqua G (1998). Spectrum of GCDFP-15 expression in human fetal and adult normal tissues. Virchows Arch.

[25] Su L, Morgan PR, Lane EB (1996). Expression of cytokeratin messenger RNA versus protein in the normal mammary gland and in breast cancer. Hum Pathol.

[26] Brotherick I, Robson CN, Browell DA, Shenfine J, White MD, Cunliffe WJ (1998). Cytokeratin expression in breast cancer: phenotypic changes associated with disease progression. Cytometry.

[27] Mi H, Muruganujan A, Ebert D, Huang X, Thomas PD (2019). PANTHER version 14: more genomes, a new PANTHER GO-slim and improvements in enrichment analysis tools. Nucleic Acids Res.

[28] Mundel P, Reiser J, Zúñiga Mejía Borja A, Pavenstädt H, Davidson GR, Kriz W (1997). Rearrangements of the cytoskeleton and cell contacts induce process formation during differentiation of conditionally immortalized mouse podocyte cell lines. Exp Cell Res.

[29] Swaminathan V, Mythreye K, O’Brien ET, Berchuck A, Blobe GC, Superfine R (2011). Mechanical stiffness grades metastatic potential in patient tumor cells and in cancer cell linesmechanical stiffness of cells dictates cancer cell invasion. Cancer Res AACR.

[30] Starodubtseva MN (2011). Mechanical properties of cells and ageing. Ageing Res Rev.

[31] Neville MC, Zhang P (2000). Lactoferrin secretion into milk: comparison between ruminant, murine, and human milk [Internet]. J Anim Sci.

[32] De Groot N, Van Kuik-Romeijn P, Lee SH, De Boer HA (2000). Increased immunoglobulin a levels in milk by over-expressing the murine polymeric immunoglobulin receptor gene in the mammary gland epithelial cells of transgenic mice. Immunology.

[33] Liu W, Rodgers GP (2016). Olfactomedin 4 expression and functions in innate immunity, inflammation, and cancer. Cancer Metastasis Rev.

[34] Maimouni S, Lee M-H, Sung Y-M, Hall M, Roy A, Ouaari C (2019). Tumor suppressor RARRES1 links tubulin deglutamylation to mitochondrial metabolism and cell survival. Oncotarget.

[35] Yokoi A, Kuronuma K, Fukuoka T, Goto C, Matsuo M, Adachi E (2018). MGP interacts with BMP-4 and BMP-2 and supports normal and malignant hematopoietic stem/progenitor cells. Blood.

[36] Virtanen S, Schulte R, Stingl J, Caldas C, Shehata M (2021). High-throughput surface marker screen on primary human breast tissues reveals further cellular heterogeneity. Breast Cancer Res.

[37] Harrell JC, Shroka TM, Jacobsen BM (2017). Estrogen induces c-Kit and an aggressive phenotype in a model of invasive lobular breast cancer. Oncogenesis.

[38] Janostiak R, Vyas M, Cicek AF, Wajapeyee N, Harigopal M (2018). Loss of c-KIT expression in breast cancer correlates with malignant transformation of breast epithelium and is mediated by KIT gene promoter DNA hypermethylation [Internet]. Exp Mol Pathol.

[39] Todhunter ME, Miyano M, Moolamalla DS, Filippov A, Sayaman RW, LaBarge MA (2021). Volume-constrained microcontainers enable myoepithelial functional differentiation in highly parallel mammary organoid culture. iScience.

[40] Giulivi C, Traaseth NJ, Davies KJA (2003). Tyrosine oxidation products: analysis and biological relevance. Amino Acids.

[41] Stampfer M, Hallowes RC, Hackett AJ (1980). Growth of normal human mammary cells in culture. In Vitro.

[42] Hammond SL, Ham RG, Stampfer MR (1984). Serum-free growth of human mammary epithelial cells: rapid clonal growth in defined medium and extended serial passage with pituitary extract. Proc Natl Acad Sci U S A.

[43] Niranjan B, Lawrence MG, Papargiris MM, Richards MG, Hussain S, Frydenberg M (2013). Primary culture and propagation of human prostate epithelial cells. Methods Mol Biol.

[44] Jiang W, Lu JQ, Yang LV, Sa Y, Feng Y, Ding J (2016). Comparison study of distinguishing cancerous and normal prostate epithelial cells by confocal and polarization diffraction imaging. J Biomed Opt.

[45] Li ML, Aggeler J, Farson DA, Hatier C, Hassell J, Bissell MJ (1987). Influence of a reconstituted basement membrane and its components on casein gene expression and secretion in mouse mammary epithelial cells. Proc Natl Acad Sci U S A.

[46] Kaushik G, Ponnusamy MP, Batra SK (2018). Concise review: current status of three-dimensional organoids as preclinical models. Stem Cells.

