# Supplementary figures and images for: Sustained postconfluent culture of human mammary epithelial cells enriches for luminal and c-Kit+ subtypes
Source: Breast Cancer Res. 2023 Jan 18;25:6. doi: 10.1186/s13058-022-01595-z (PMC9847146; doi:10.1186/s13058-022-01595-z)

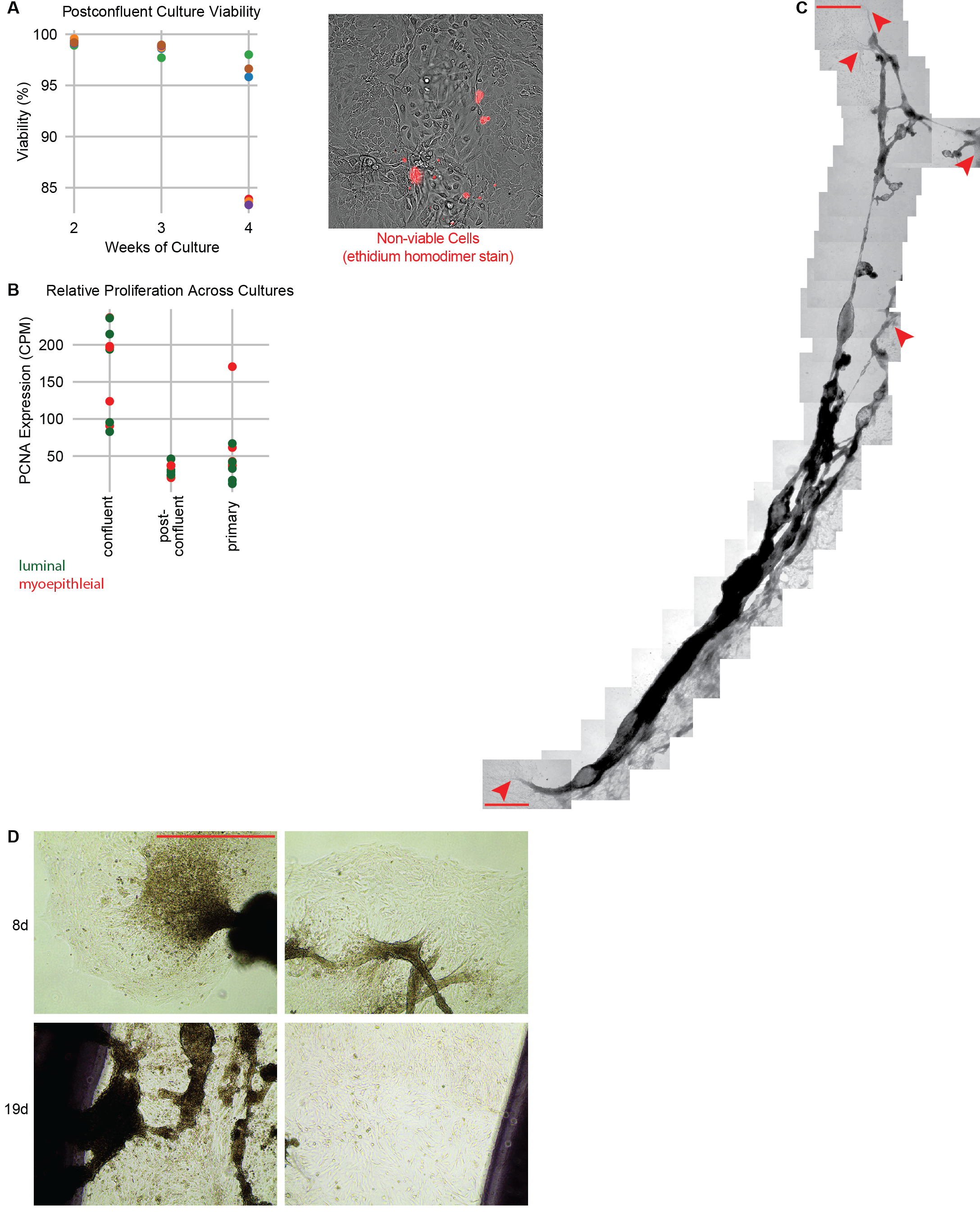

Supplement: Supplementary file 2 — Additional file 2: Fig. S1. A Viability of postconfluent cultures over time, as measured by ethidium homodimer incorporation. Each color indicates an independent HMEC specimen (n = 6). Estimated marginal mean post hoc analysis for a repeated measures linear model of logit-transformed percent viability (see Methods) confirms that the viability of postconfluent cultures does not significantly drop until week four, with p < 0.001. B Proliferation of postconfluent cultures, as measured by proliferating cell nuclear antigen (PCNA). Tukey’s HSD post hoc analysis confirms that counts-per-million PCNA gene expression significantly drops from confluent to postconfluent culture (p < 0.001), but postconfluent expression is not significantly different from primary expression. C Macroscopic structure from postconfluent HMEC culture on 2 kPa collagen-coated polyacrylamide. Attachment points to substrate denoted with red arrowheads. D Advanced time points for postconfluent structures detached from plastic with collagenase, then reattached and grown out on fresh dishes. Columns are replicates. The black regions visible in the 19d time point are fiducial ink markings drawn on the underside of the dish. Scale bars are 1 mm. [file 13058_2022_1595_MOESM2_ESM.png]

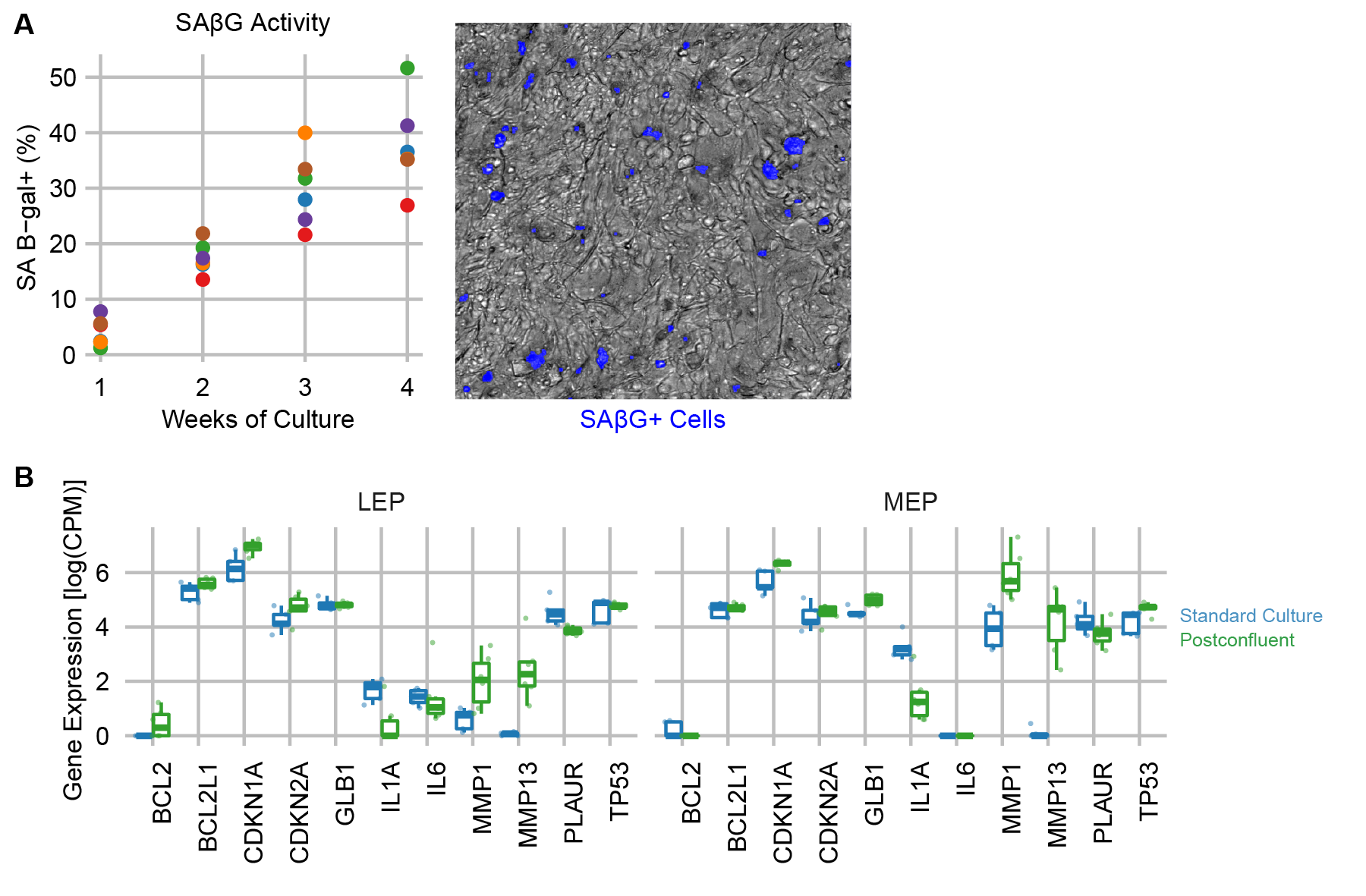

Supplement: Supplementary file 3 — Additional file 3: Fig. S3. A Senescence of postconfluent cultures over time, as measured by area fraction of cells senescence-associated β-galactosidase activity (SABG). Each color indicates an independent HMEC specimen (n = 6). Estimated marginal mean post hoc analysis for a repeated measures linear model of logit-transformed percent SABG positive cells (the Methods) confirms that the senescence of postconfluent cultures rises over time, with p < 0.001. B Relative gene expression for characteristic senescence-associated genes of FACS-sorted LEps and MEps from three-week postconfluent culture. [file 13058_2022_1595_MOESM3_ESM.png]

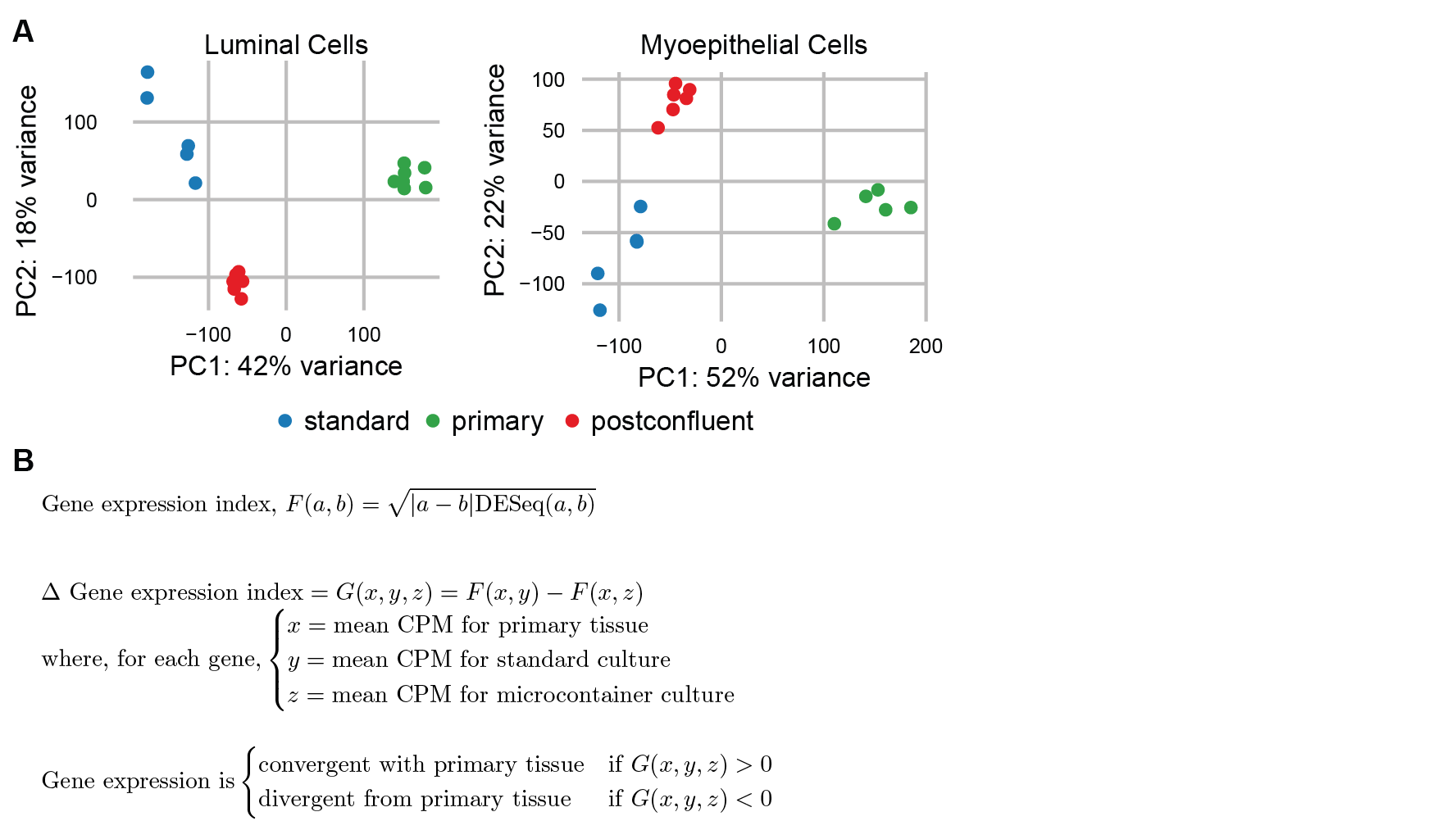

Supplement: Supplementary file 8 — Additional file 8: Fig. S2. A Principal component analysis of culture conditions, divided by lineage. B Equation for differential expression index. [file 13058_2022_1595_MOESM8_ESM.png]
